# Supplementary material for: Comparison of SYK Signaling Networks Reveals the Potential Molecular Determinants of Its Tumor-Promoting and Suppressing Functions
Source: Biomolecules. 2021 Feb 18;11(2):308. doi: 10.3390/biom11020308 (PMC7923165; doi:10.3390/biom11020308)
Supplement: Supplementary file 1 [file biomolecules-11-00308-s001.zip › Buffard&Larive_Supplementary-Material-complete/Buffard&Larive_Supplementary-Figures.pdf]

Buffard et al.

Figure S1

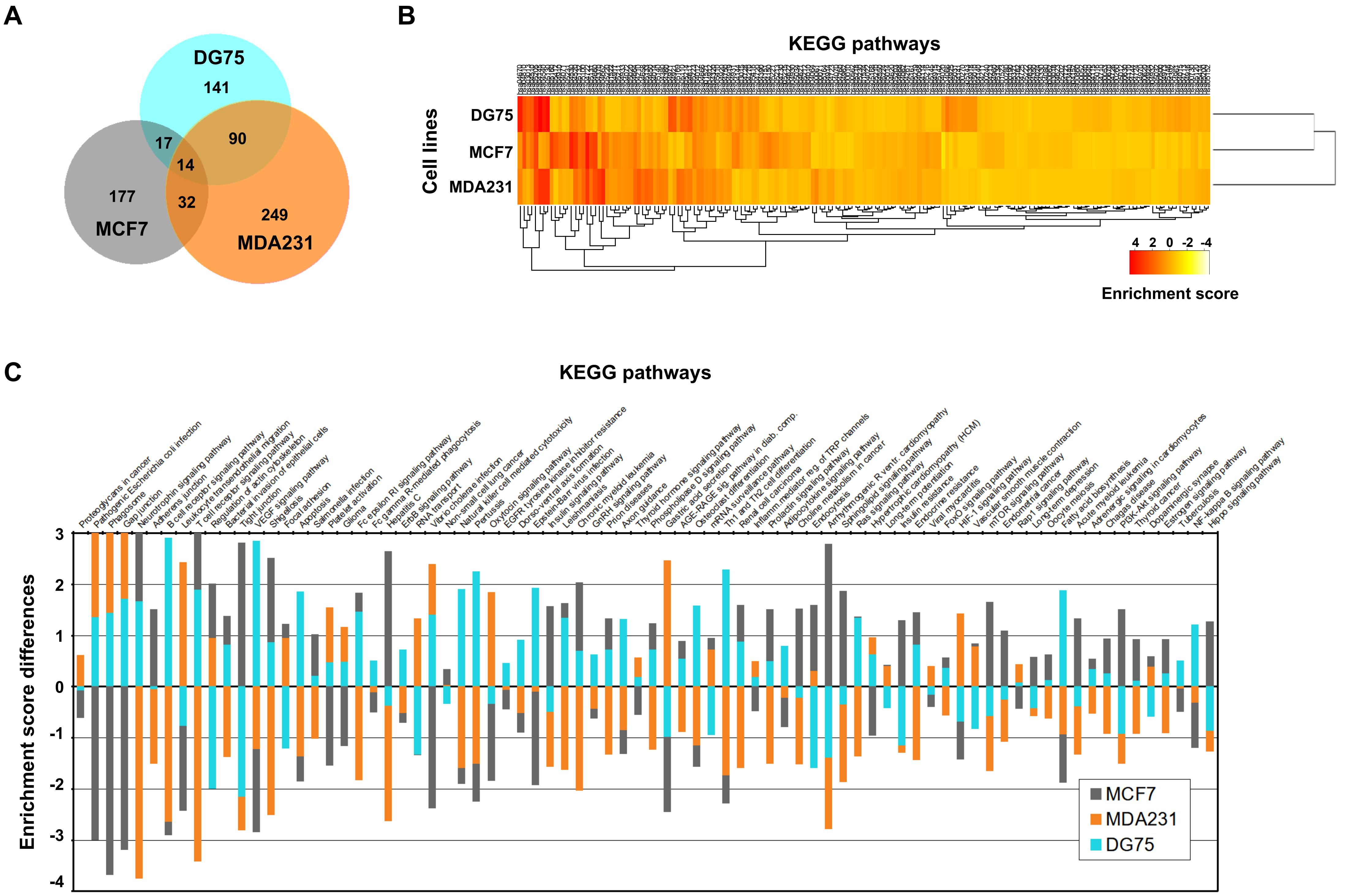

Proteins not identified in the datasets :

Proteins identified in the dataset : MCF7 | MDA231 | DG75 | MDA231 & DG75 | MCF7 & DG75 | MCF7, MDA231 & DG75

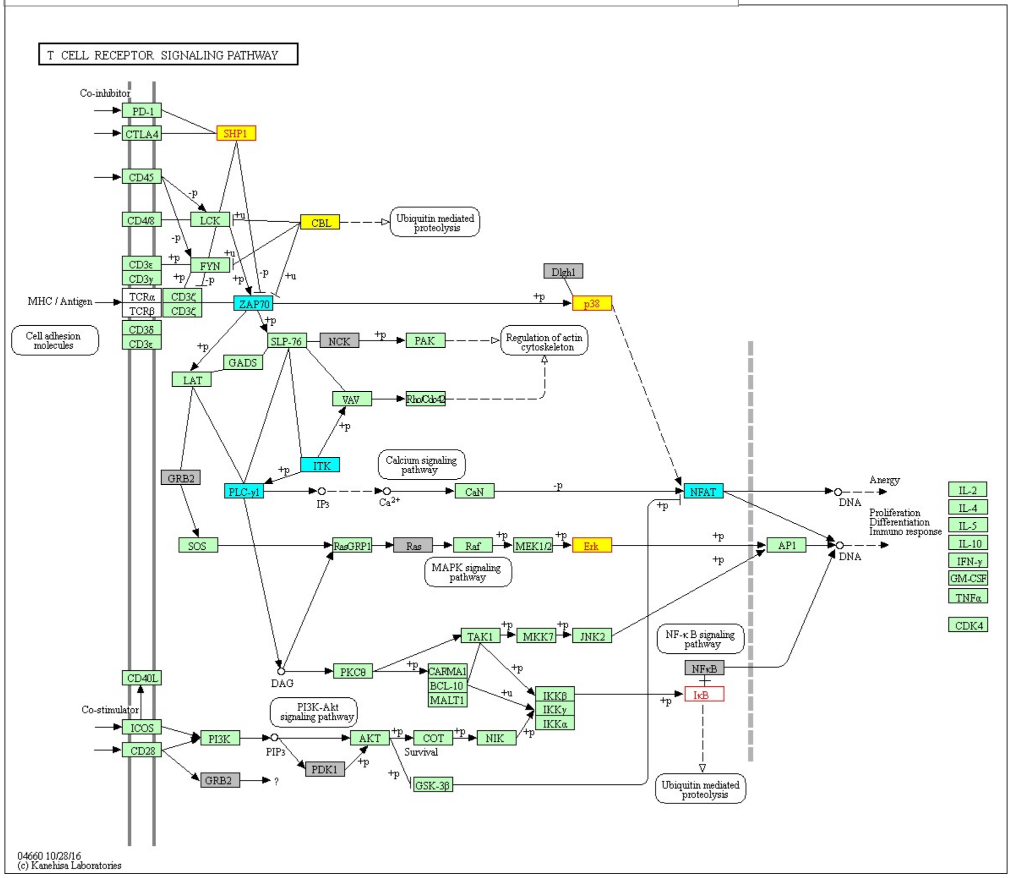

hsa04660 [['PTPN6', 'MAPK14', 'PDPK1', 'NRAS', 'GRB2', 'RELA', 'NCK1', 'DLG1', 'MAPK1', 'CBL'], ['NFKBIE', 'CBL'], ['NFKBIE', 'NFATC2', 'PLCG1', 'PTPN6', 'TEC', 'MAPK14', 'MAPK13', 'MAPK3', 'ZAP70', 'MAPK1']]

Uniprot	KEGG	Symbol	Value	MDA D	MDA sites	DG75 D	DG75 sites
O00221	4794	NFKBIE	-BC		155		155
Q13469	4773	NFATC2	--C				752
P19174	5335	PLCG1	--C				771;775;783
P29350	5777	PTPN6	A-C				536
P42680	7006	TEC	--C				519
Q16539	1432	MAPK14	A-C				182
O15530	5170	PDPK1	A--				
P01111	4893	NRAS	A--				
P62993	2885	GRB2	A--				
O15264	5603	MAPK13	--C				182
P27361	5595	MAPK3	--C				204
Q04206	5970	RELA	A--				
P16333	4690	NCK1	A--				
Q12959	1739	DLG1	A--				
P43403	7535	ZAP70	--C				292
P28482	5594	MAPK1	A-C				187
P22681	867	CBL	AB-		700		

BUFFARD et al.

Figure S3

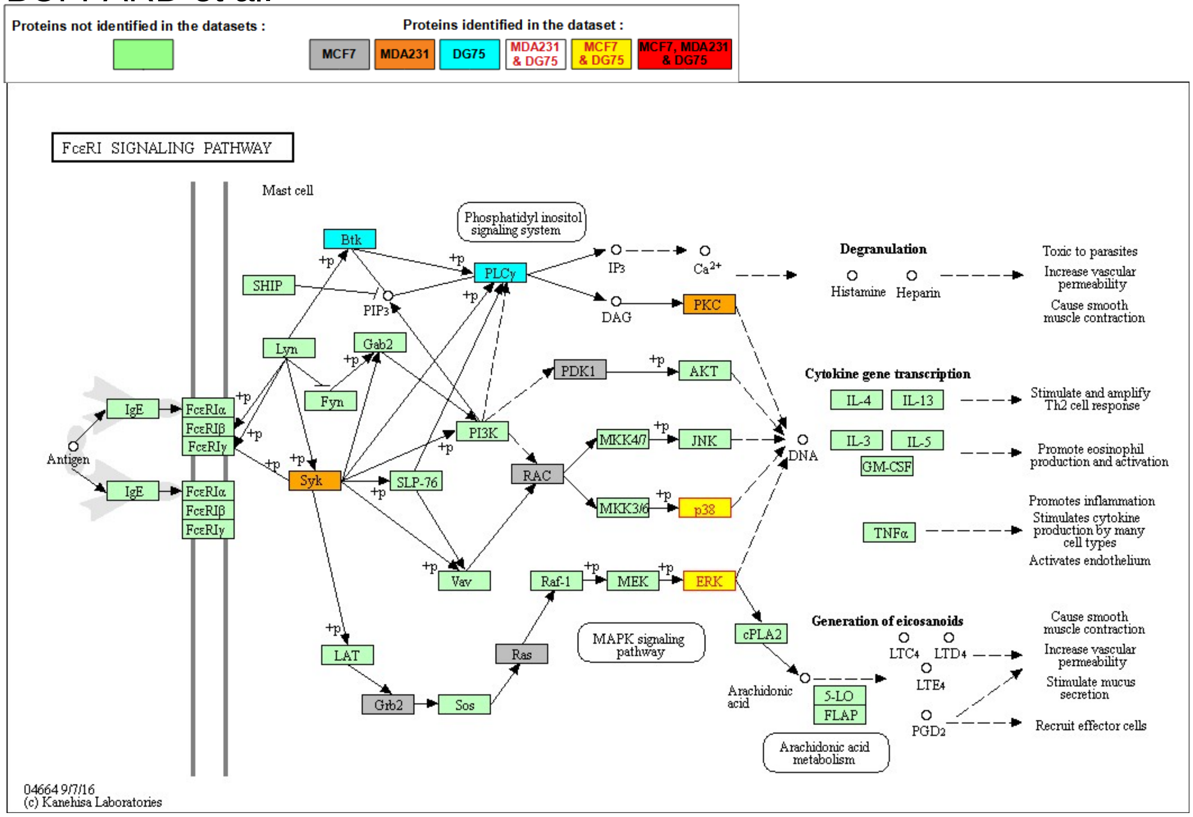

hsa04664 [['MAPK14', 'PDPK1', 'RAC1', 'NRAS', 'GRB2', 'MAPK1'], ['SYK', 'PRKCA', 'PLCG2'], ['PLCG1', 'MAPK14', 'MAPK13', 'PLCG2', 'BTK', 'MAPK3', 'MAPK1']]

Uniprot	KEGG	Symbol	Value	MDA D	MDA sites	DG75 D	DG75 sites
P19174	5335	PLCG1	--C				771;775;783
Q16539	1432	MAPK14	A-C				182
P01111	4893	NRAS	A--				
O15530	5170	PDPK1	A--				
P63000	5879	RAC1	A--				
P16885	5336	PLCG2	-BC		1217	x	759*;550;858;1245
P62993	2885	GRB2	A--				
O15264	5603	MAPK13	--C				182
Q06187	695	BTK	--C			x	551*
P43405	6850	SYK	-B-		352		
P27361	5595	MAPK3	--C				204
P28482	5594	MAPK1	A-C				187
P17252	5578	PRKCA	-B-		504		

BUFFARD et al

Figure S4

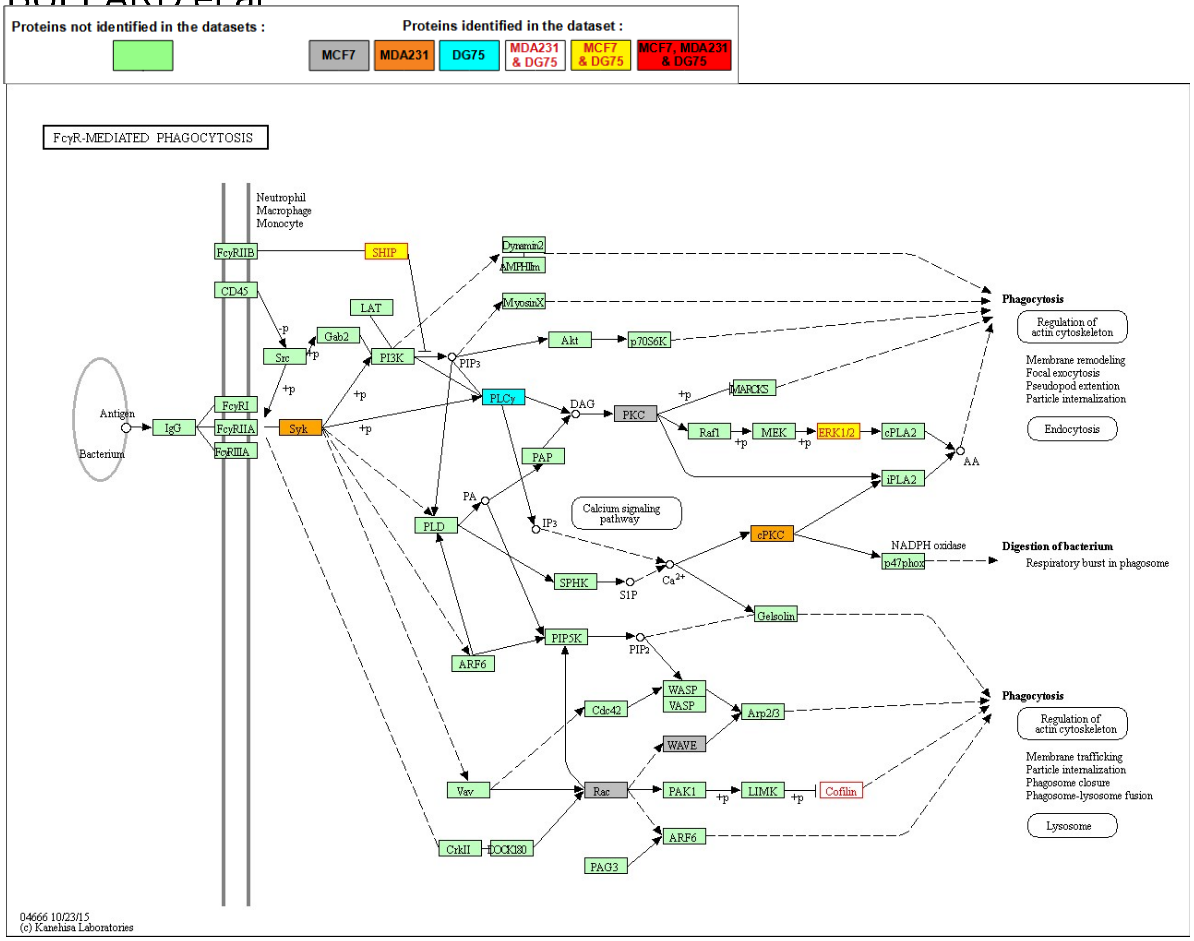

hsa04666 [['WASF2', 'RAC1', 'WASF1', 'INPPL1', 'MAPK1', 'PRKCD'], ['PRKCG', 'PLCG2', 'CFL2', 'SYK', 'PRKCB', 'PRKCA', 'CFL1'], ['PLCG1', 'PLCG2', 'CFL2', 'MAPK3', 'INPPL1', 'MAPK1', 'CFL1']]

Uniprot	KEGG	Symbol	Value	MDA D	MDA sites	DG75 D	DG75 sites
P19174	5335	PLCG1	--C				771;775;783
Q9Y6W5	10163	WASF2	A--				
P05129	5582	PRKCG	-B-		521		
P63000	5879	RAC1	A--				
P16885	5336	PLCG2	-BC		1217	x	759*;550;858;1245
Q9Y281	1073	CFL2	-BC	x	89*		89
P27361	5595	MAPK3	--C				204
P43405	6850	SYK	-B-		352		
Q92558	8936	WASF1	A--				
O15357	3636	INPPL1	A-C				1135
P05771	5579	PRKCB	-B-		507		
P23528	1072	CFL1	-BC	x	89*		68;89
P28482	5594	MAPK1	A-C				187
P17252	5578	PRKCA	-B-		504		
Q05655	5580	PRKCD	A--				

BUFFARD et al. Figure S5

Proteins not identified in the datasets : (green)

Proteins identified in the dataset : MCF7 | MDA231 | DG75 | MDA231 & DG75 | MCF7 & DG75 | MCF7, MDA231 & DG75

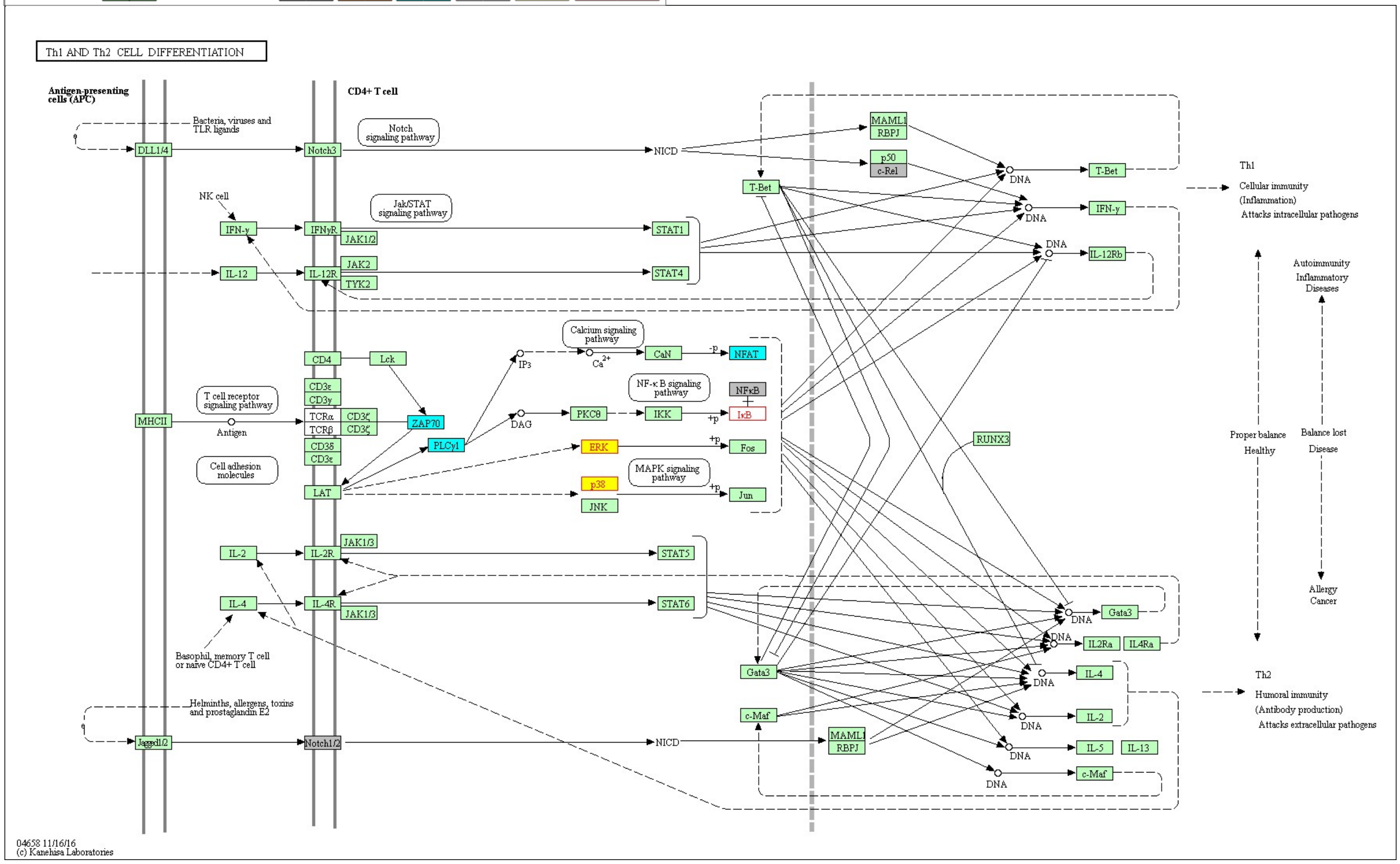

hsa04658 [['NOTCH2', 'MAPK1', 'MAPK14', 'RELA'], ['NFKBIE'], ['NFKBIE', 'PLCG1', 'NFATC2', 'MAPK14', 'MAPK13', 'MAPK3', 'ZAP70', 'MAPK1']]

Uniprot	KEGG	Symbol	Value	MDA D	MDA sites	DG75 D	DG75 sites
O00221	4794	NFKBIE	-BC		155		155
P19174	5335	PLCG1	--C				771;775;783
Q13469	4773	NFATC2	--C				752
Q16539	1432	MAPK14	A-C				182
O15264	5603	MAPK13	--C				182
Q04721	4853	NOTCH2	A--				
Q04206	5970	RELA	A--				
P27361	5595	MAPK3	--C				204
P43403	7535	ZAP70	--C				292
P28482	5594	MAPK1	A-C				187

BUFFARD et al. Figure S6

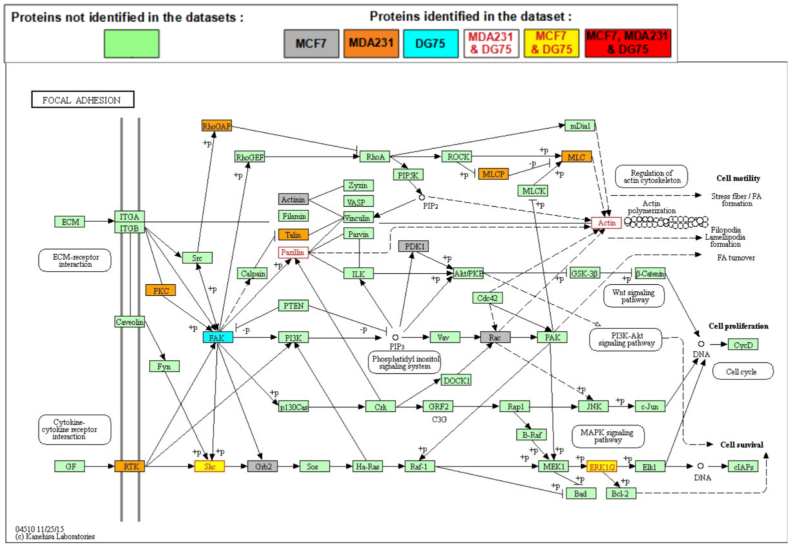

hsa04510 [['SHC1', 'PDPK1', 'PPP1CA', 'RAC1', 'ACTN1', 'GRB2', 'MYL12A', 'ACTN4', 'PPP1CB', 'MAPK1'], ['PXN', 'MYL12B', 'PRKCG', 'TLN1', 'PPP1R12A', 'PRKCB', 'MYL12A', 'EGFR', 'ARHGAP35', 'ACTB', 'ACTG1', 'PRKCA'], ['PXN', 'SHC1', 'MAPK3', 'MAPK1', 'ACTB', 'ACTG1', 'PTK2']]

Uniprot	KEGG	Symbol	Value	MDA D	MDA sites	DG75 D	DG75 sites
P49023	5829	PXN	-BC		409;468		88
P29353	6464	SHC1	A-C				427
O14950	103910	MYL12B	-B-		143		
Q9Y490	7094	TLN1	-B-		127		
P12814	87	ACTN1	A--				
O14974	4659	PPP1R12A	-B-		446;446		
Q05397	5747	PTK2	--C				861
P05771	5579	PRKCB	-B-		507		
P62140	5500	PPP1CB	A--				
P28482	5594	MAPK1	A-C				187
P60709	60	ACTB	-BC		240		240
P05129	5582	PRKCG	-B-		521		
O15530	5170	PDPK1	A--				
P62136	5499	PPP1CA	A--				
P63000	5879	RAC1	A--				
P62993	2885	GRB2	A--				
P27361	5595	MAPK3	--C				204
P19105	10627	MYL12A	AB-		142		
P00533	1956	EGFR	-B-		1069		
O43707	81	ACTN4	A--				
Q9NRY4	2909	ARHGAP35	-B-		1087		
P63261	71	ACTG1	-BC		240		240
P17252	5578	PRKCA	-B-		504		

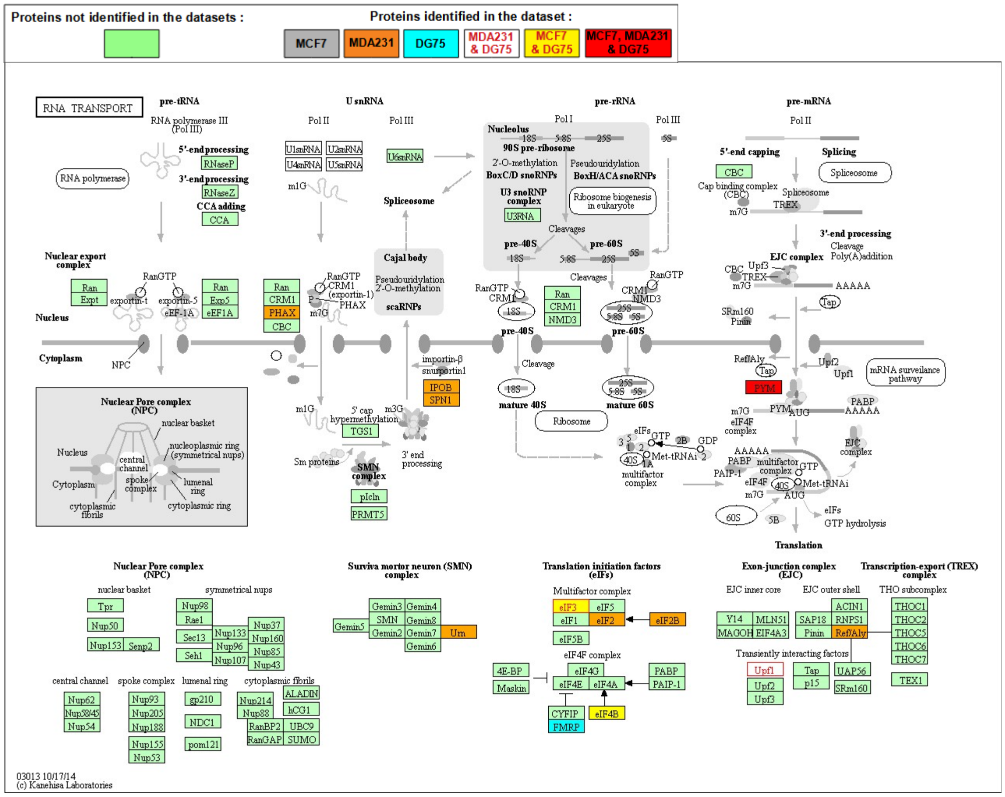

hsa03013 [['EIF3A', 'EIF3G', 'EIF4B', 'WIBG', 'EIF3F', 'EIF3I', 'EIF3E', 'EIF3J'], ['STRAP', 'SNUPN', 'PHAX', 'UPF1', 'ALYREF', 'EIF4B', 'EIF2S1', 'WIBG', 'KPNB1', 'EIF3J', 'EIF2B1'], ['FXR1', 'EIF3E', 'WIBG', 'FXR2', 'UPF1']]

Uniprot	KEGG	Symbol	Value	MDA D	MDA sites	DG75 D	DG75 sites
Q9H814	51808	PHAX	-B-		57		
Q92900	5976	UPF1	-BC		1112;113		113;1112
Q14152	8661	EIF3A	A--				
P23588	1975	EIF4B	AB-		211;609		
Q9BRP8	84305	WIBG	ABC	x	45*		45
O00303	8665	EIF3F	A--				
O75822	8669	EIF3J	AB-		254		
Q14232	1967	EIF2B1	-B-		83		
O95149	10073	SNUPN	-B-		334		
Q86V81	10189	ALYREF	-B-		250		
P51116	9513	FXR2	--C				614
O75821	8666	EIF3G	A--				
P05198	1965	EIF2S1	-B-		200		
P51114	8087	FXR1	--C				68
Q13347	8668	EIF3I	A--				
P60228	3646	EIF3E	A-C				445
Q9Y3F4	11171	STRAP	-B-		114		
Q14974	3837	KPNB1	-B-		752;529		

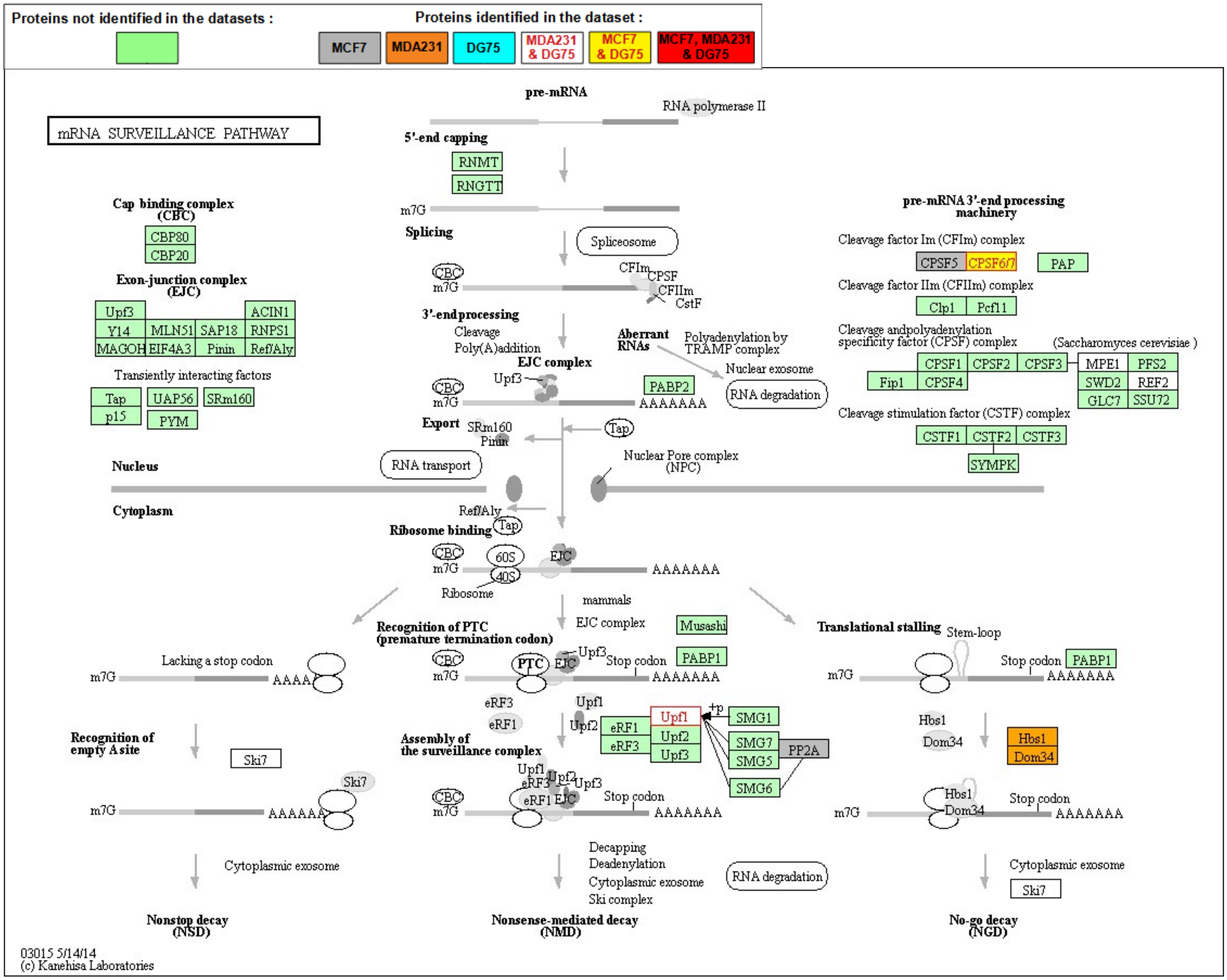

hsa03015 [['CPSF6', 'NUDT21', 'PPP2CA', 'PPP2R1A'], ['CPSF7', 'PPP2R5D', 'UPF1', 'PELO', 'HBS1L'], ['CPSF6', 'UPF1']]

Uniprot	KEGG	Symbol	Value	MDA D	MDA sites	DG75 D	DG75 sites
Q16630	11052	CPSF6	A-C				74
Q14738	5528	PPP2R5D	-B-		580		
Q92900	5976	UPF1	-BC		1112;113		113;1112
P30153	5518	PPP2R1A	A--				
Q9Y450	10767	HBS1L	-B-		58		
Q9BRX2	53918	PELO	-B-		99		
P67775	5515	PPP2CA	A--				
Q8N684	79869	CPSF7	-B-		451		
O43809	11051	NUDT21	A--				

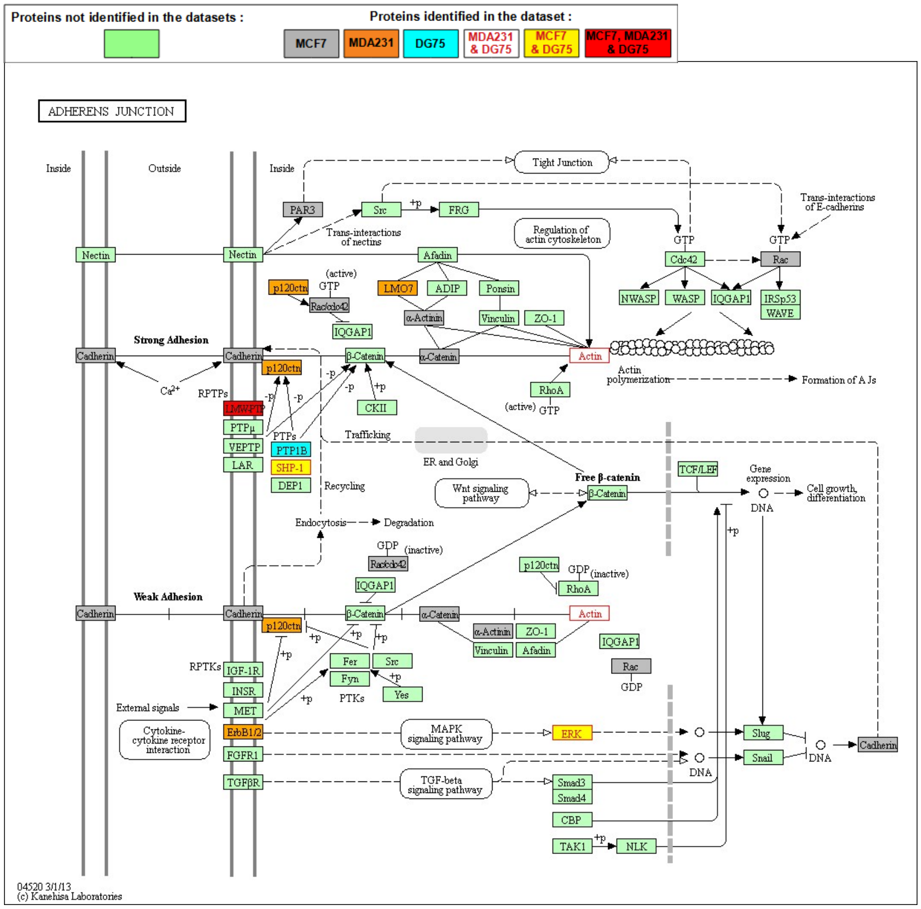

hsa04520 [['CTNNA1', 'PTPN6', 'PARD3', 'RAC1', 'ACTN1', 'ACP1', 'CDH1', 'ACTN4', 'MAPK1'], ['CTNND1', 'ACP1', 'EGFR', 'ACTB', 'ACTG1', 'LMO7'], ['PTPN6', 'MAPK3', 'ACP1', 'PTPN1', 'MAPK1', 'ACTB', 'ACTG1']]

Uniprot	KEGG	Symbol	Value	MDA D	MDA sites	DG75 D	DG75 sites
P35221	1495	CTNNA1	A--				
O60716	1500	CTNND1	-B-		334		
P29350	5777	PTPN6	A-C				536
Q8TEW0	56288	PARD3	A--				
P63000	5879	RAC1	A--				
P12814	87	ACTN1	A--				
P27361	5595	MAPK3	--C				204
P24666	52	ACP1	ABC		120;143;50		50
P12830	999	CDH1	A--				
P00533	1956	EGFR	-B-		1069		
P18031	5770	PTPN1	--C				66
O43707	81	ACTN4	A--				
P28482	5594	MAPK1	A-C				187
P60709	60	ACTB	-BC		240		240
P63261	71	ACTG1	-BC		240		240
Q8WWI1	4008	LMO7	-B-		185;185		

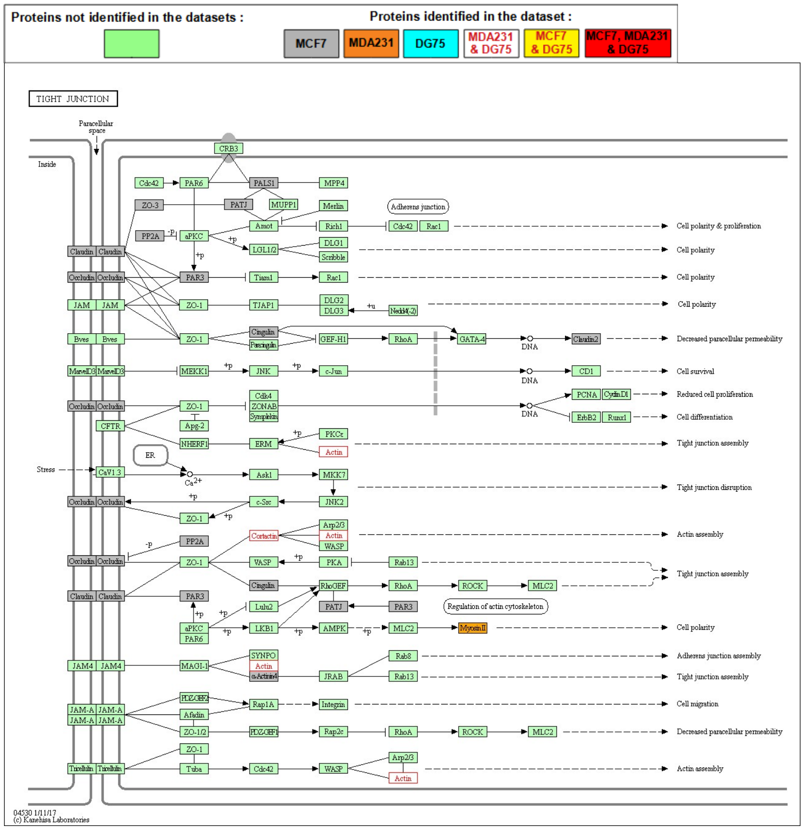

hsa04530 [['CTNNA1', 'OCLN', 'PPP2R1A', 'CLDN3', 'NRAS', 'PARD3', 'ACTN1', 'ACTN4', 'SPTAN1', 'MYL12A', 'CGN', 'PPP2CA', 'INADL', 'MPP5', 'TJP3', 'PRKCD'], ['PRKCG', 'MYL12B', 'CTTN', 'PRKCB', 'MYL12A', 'EPB41L2', 'ACTB', 'ACTG1', 'PRKCA'], ['HCLS1', 'CTTN', 'ACTB', 'ACTG1', 'EPB41L2']]

Uniprot	KEGG	Symbol	Value	MDA D	MDA sites	DG75 D	DG75 sites
O14950	103910	MYL12B	-B-		143		
P30153	5518	PPP2R1A	A--				
P63261	71	ACTG1	-BC		240		240
P01111	4893	NRAS	A--				
P12814	87	ACTN1	A--				
Q8NI35	10207	INADL	A--				
P05771	5579	PRKCB	-B-		507		
P67775	5515	PPP2CA	A--				
O43491	2037	EPB41L2	-BC		773		773
Q8N3R9	64398	MPP5	A--				
P60709	60	ACTB	-BC		240		240
Q05655	5580	PRKCD	A--				
P35221	1495	CTNNA1	A--				
Q16625	100506658	OCLN	A--				
P05129	5582	PRKCG	-B-		521		
O15551	1365	CLDN3	A--				
Q8TEW0	56288	PARD3	A--				
Q14247	2017	CTTN	-BC		433;334		446
P19105	10627	MYL12A	AB-		142		
Q9P2M7	57530	CGN	A--				
O43707	81	ACTN4	A--				
Q13813	6709	SPTAN1	A--				
P14317	3059	HCLS1	--C			x	140*;222*;103*
O95049	27134	TJP3	A--				
P17252	5578	PRKCA	-B-		504		

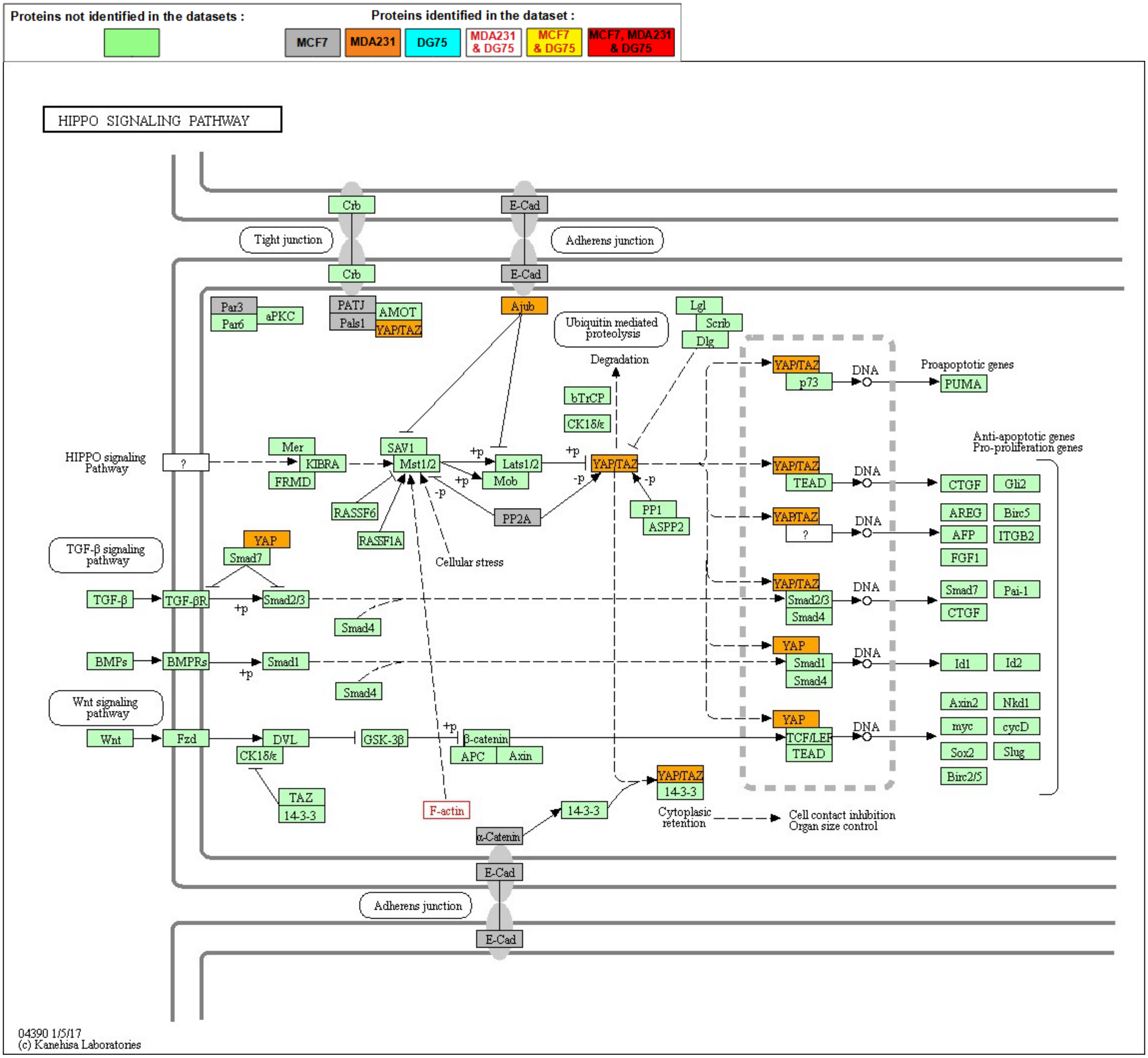

hsa04390 [['CTNNA1', 'PPP2R1A', 'PARD3', 'CDH1', 'PPP2CA', 'INADL', 'MPP5'], ['YAP1', 'LIMD1', 'ACTG1', 'ACTB'], ['ACTB', 'ACTG1']]

Uniprot	KEGG	Symbol	Value	MDA D	MDA sites	DG75 D	DG75 sites
P35221	1495	CTNNA1	A--				
P46937	10413	YAP1	-B-		407;391		
P30153	5518	PPP2R1A	A--				
Q8TEW0	56288	PARD3	A--				
P60709	60	ACTB	-BC		240		240
P12830	999	CDH1	A--				
P67775	5515	PPP2CA	A--				
Q8NI35	10207	INADL	A--				
Q8N3R9	64398	MPP5	A--				
Q9UGP4	8994	LIMD1	-B-		179		
P63261	71	ACTG1	-BC		240		240

Buffard et al.

Figure S12

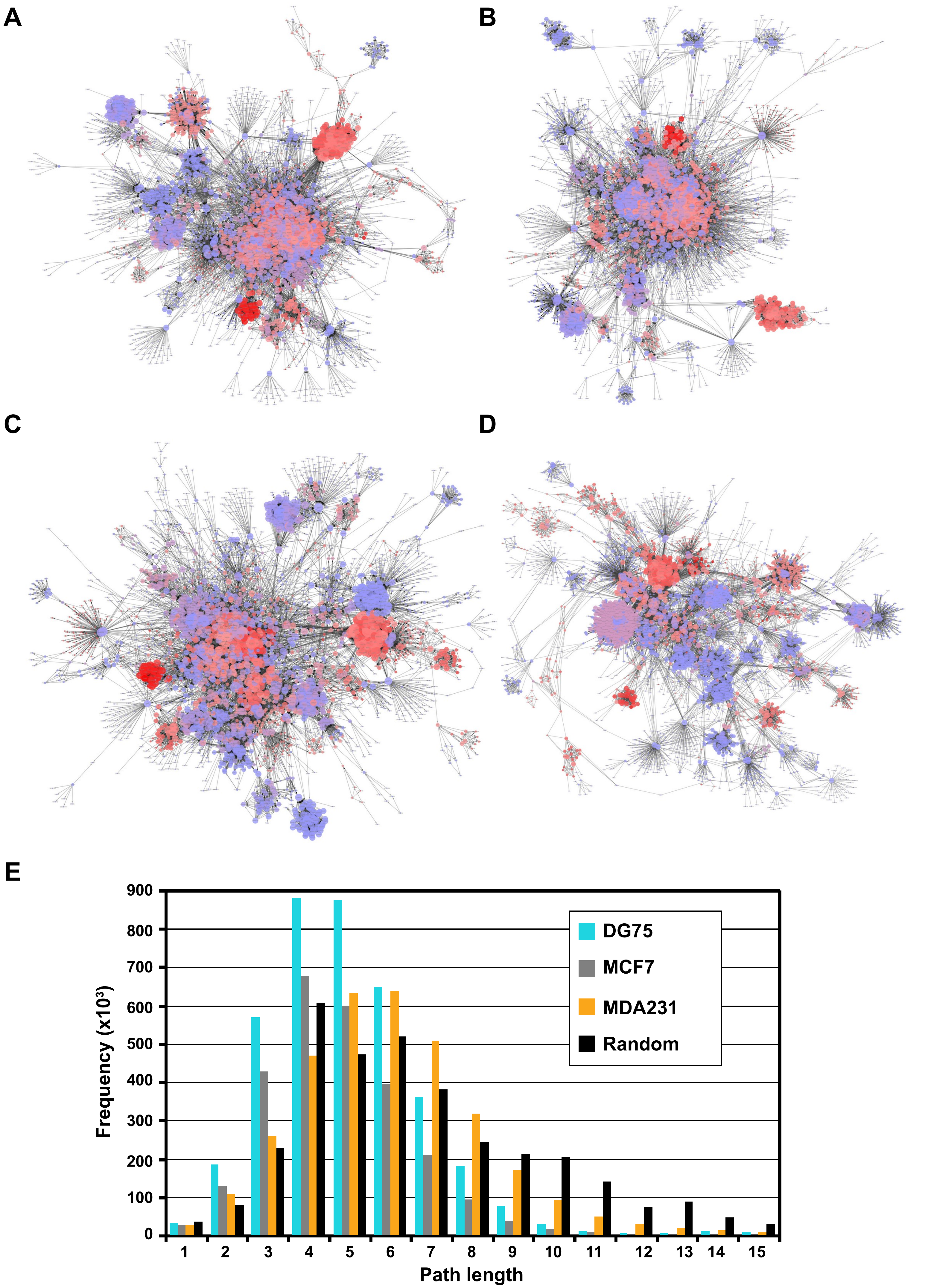

Buffard et al.

Figure S13

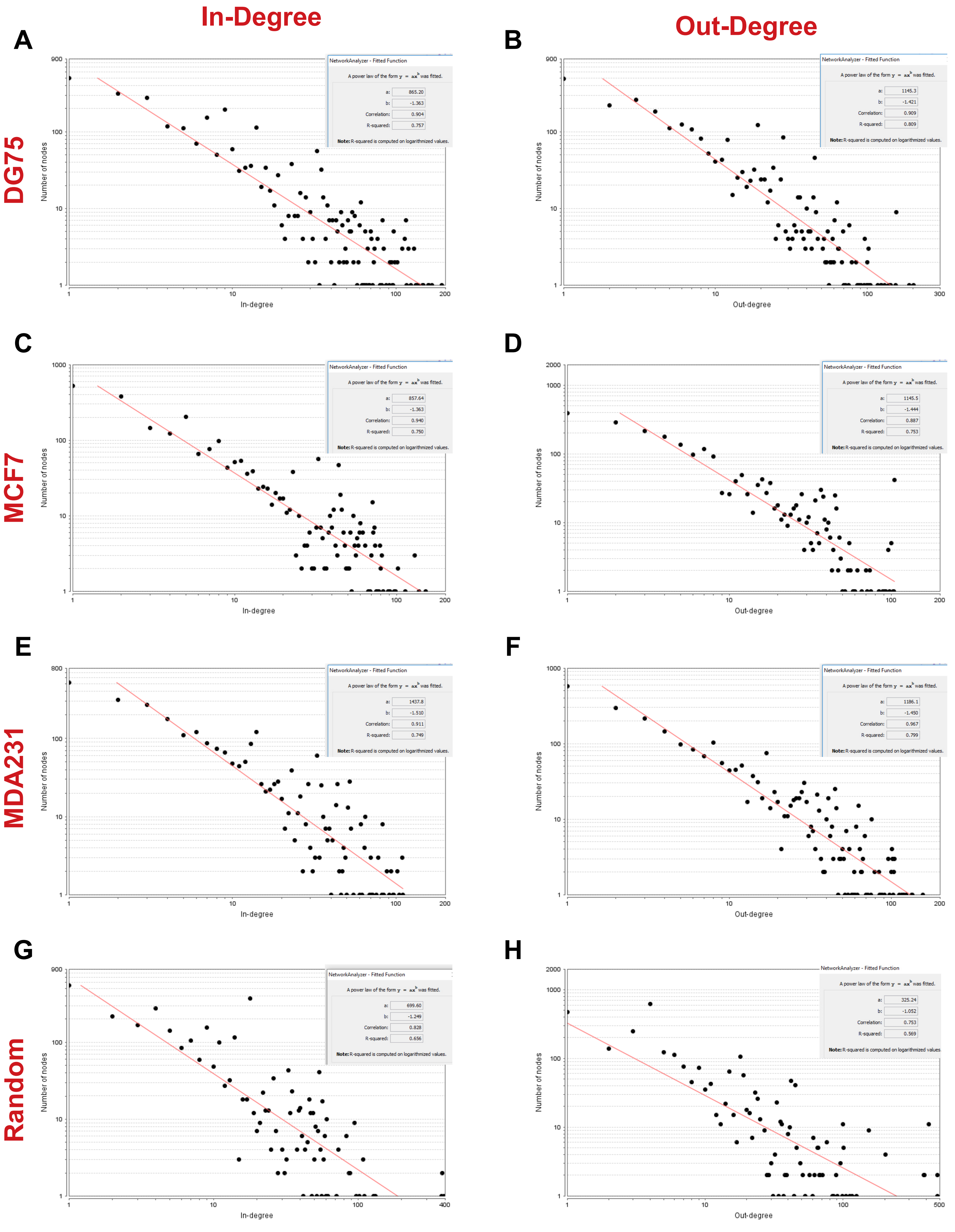

Table S3. Topological parameters of the protein networks

	DG75	MCF7	MDA231	Random
Number of protein inputs	364	240	387	294
Number of nodes	3417	3336	3440	3807
Number of edges	33525	28814	29685	37565
In-degree scaling index (power law exponent)	1.363	1.363	1.510	1,249
In-degree : Power law correlation	0.904	0.940	0.911	0,828
Out-degree scaling index (power law exponent)	1.421	1.444	1.450	1,052
Out-degree : Power law correlation	0.909	0.887	0.967	0,753
Characteristic path lengh	5.017	4.771	5.977	6,692

Table S4. Centrality parameters of SYK nodes in the protein networks

	DG75	MCF7	MDA231	Random
Edge count	65	55	26	18
Indegree	19	20	5	3
Outdegree	46	35	21	15
Neighborhood connectivity	77.094	47.537	53.731	38,277
Avg. shortest path length	3.581	3.452	4.149	5,779
Betweenness centrality[1]	1.304E-3	9.734E-4	8.796E-4	2.375E-4
Closeness centrality[2]	0.279	0.290	0.241	0,173

[1] The betweenness centrality of a node reflects the amount of control that this node exerts over the interactions of other nodes in the network (Yoon, 2006).
[2] The closeness centrality is a measure of how quickly information spreads from a given node to other reachable nodes in the network (Freeman, 1979).

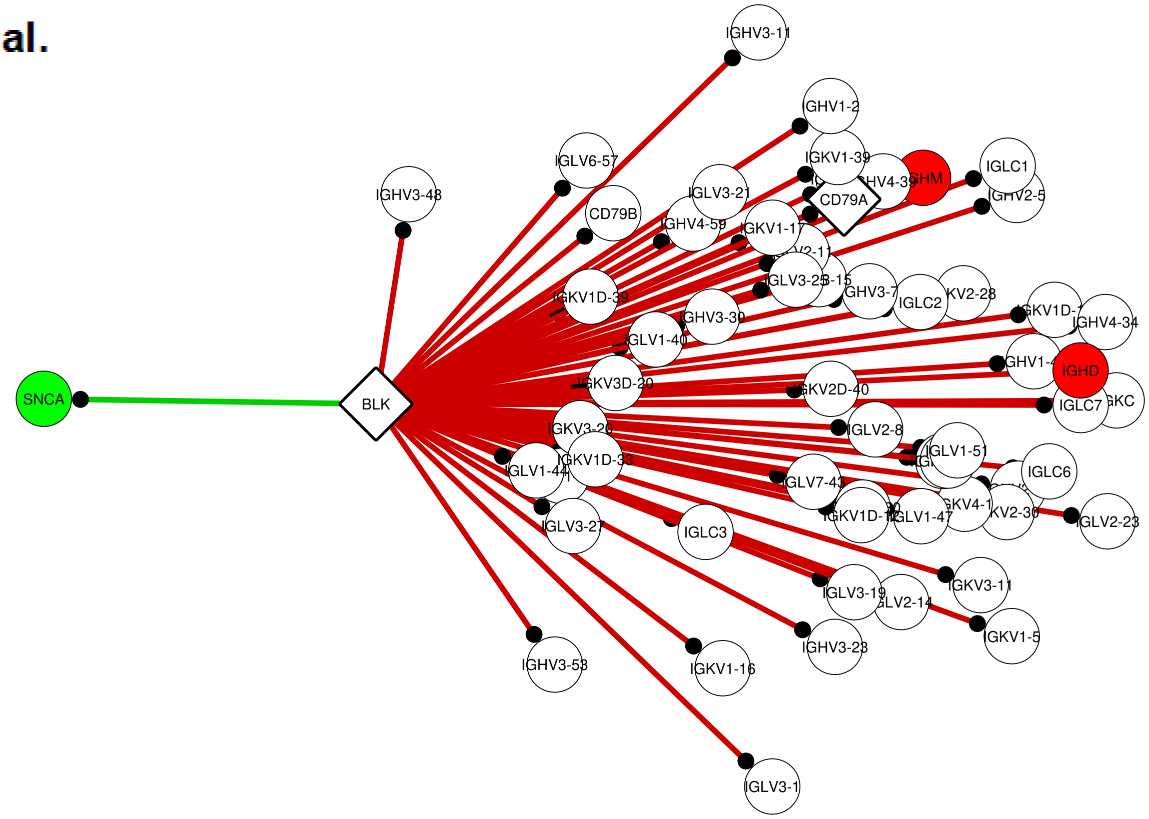
SNCA
BLK
IGHV3-11
IGHV1-2
IGKV1-39
IGHM
IGLC1
IGLV6-57
IGHV3-48
CD79B
IGLV3-21
CD79A
IGHV4-59
IGKV1-17
IGKV1D-39
IGLV3-25
IGHV3-30
IGLC2
IGLV1-40
IGHV4-34
IGHD
IGKV3D-20
IGKV2D-40
IGLC7
IGKV3-20
IGLV2-8
IGLV1-51
IGLV1-44
IGKV1D-33
IGLC6
IGLV7-43
IGLV1-47
IGLV2-23
IGLV3-27
IGLC3
IGKV3-11
IGLV3-19
IGKV1-5
IGHV3-53
IGKV1-16
IGHV3-23
IGLV3-1

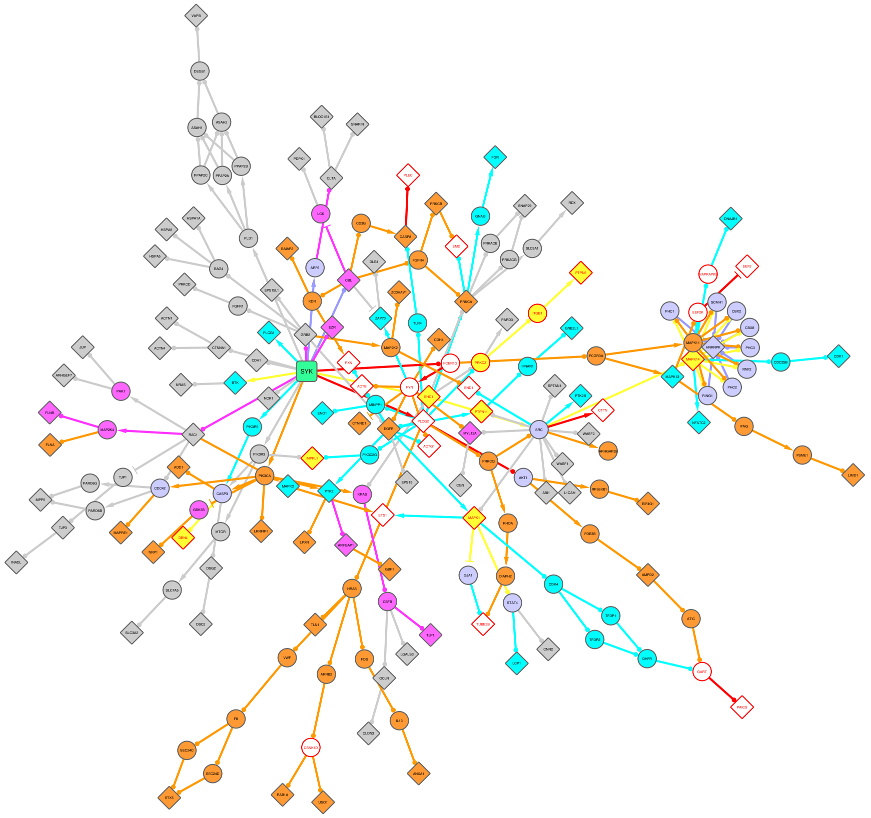

Signal propagation from Syk to its targets involved in cell adhesion and motility. (prefused force directed layout)

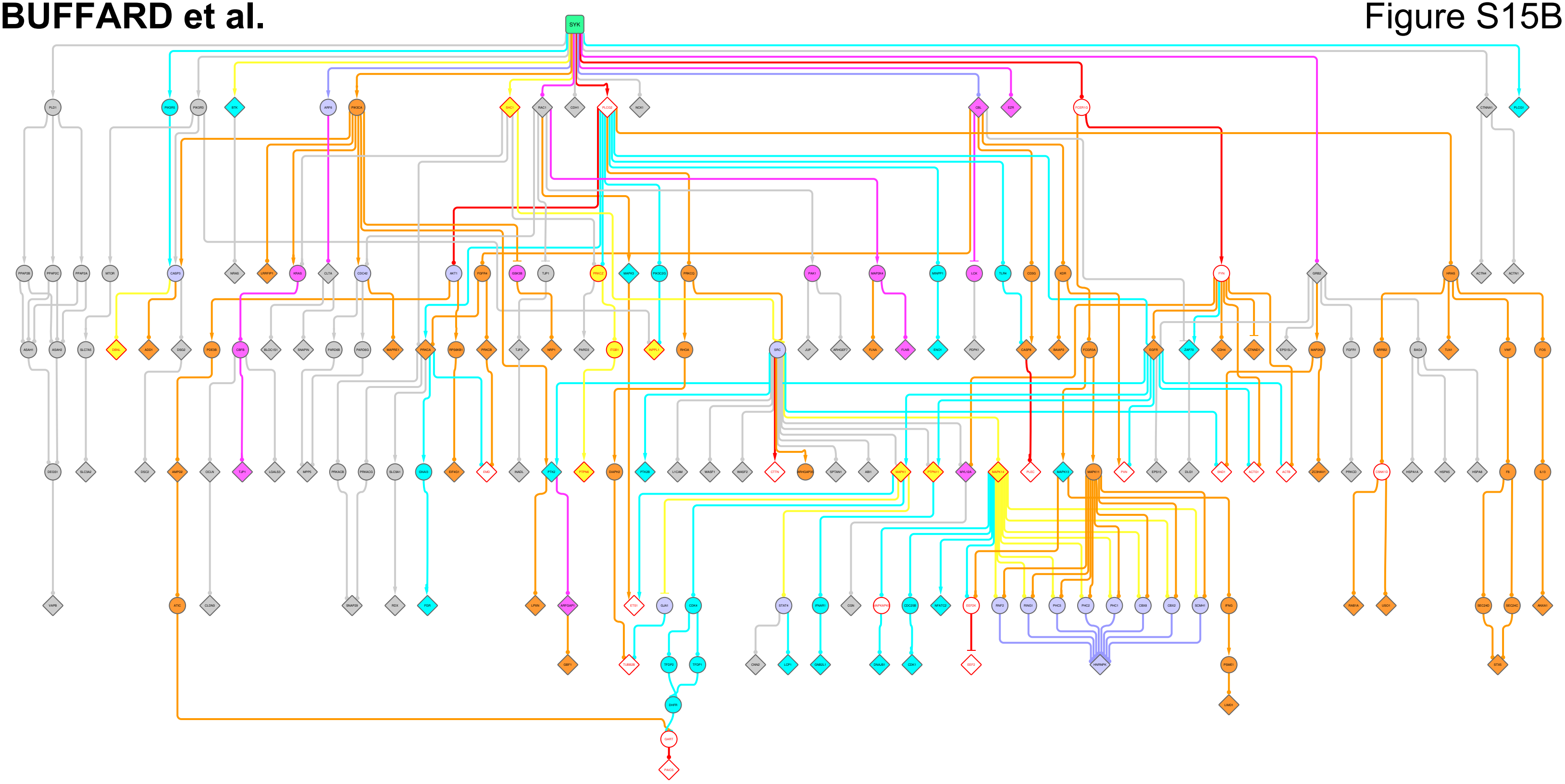

Signal propagation from Syk to its targets involved in cell adhesion and motility. (hierarchical layout)

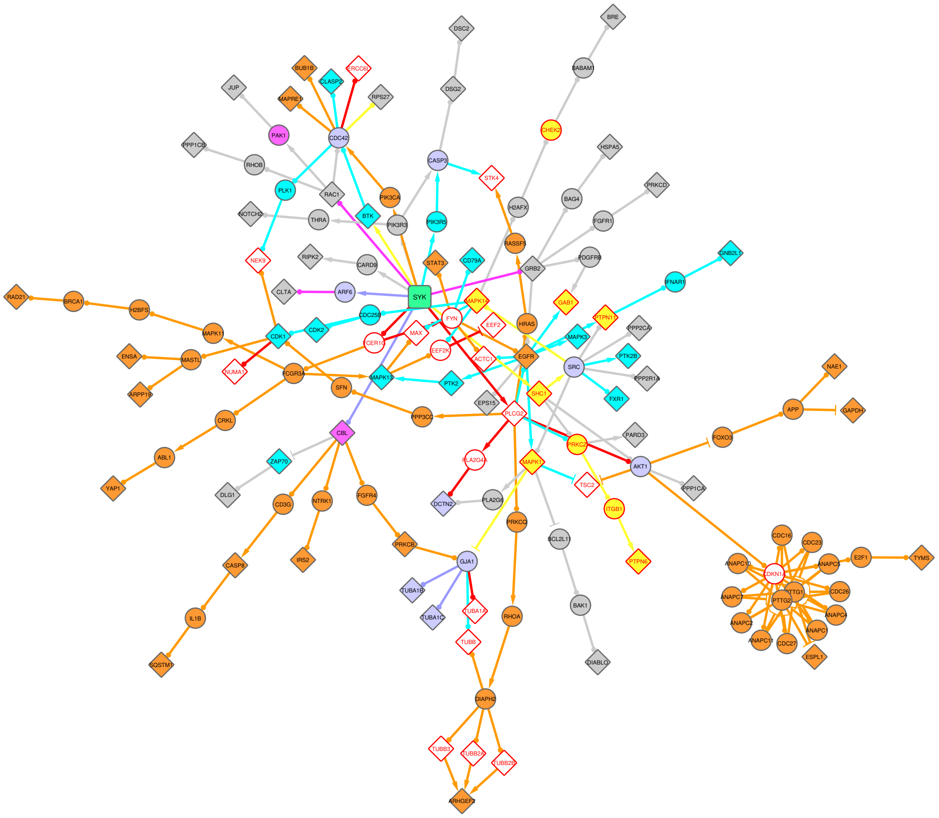

Signal propagation from Syk to its targets involved in cell growth and death.
(prefused force directed layout)

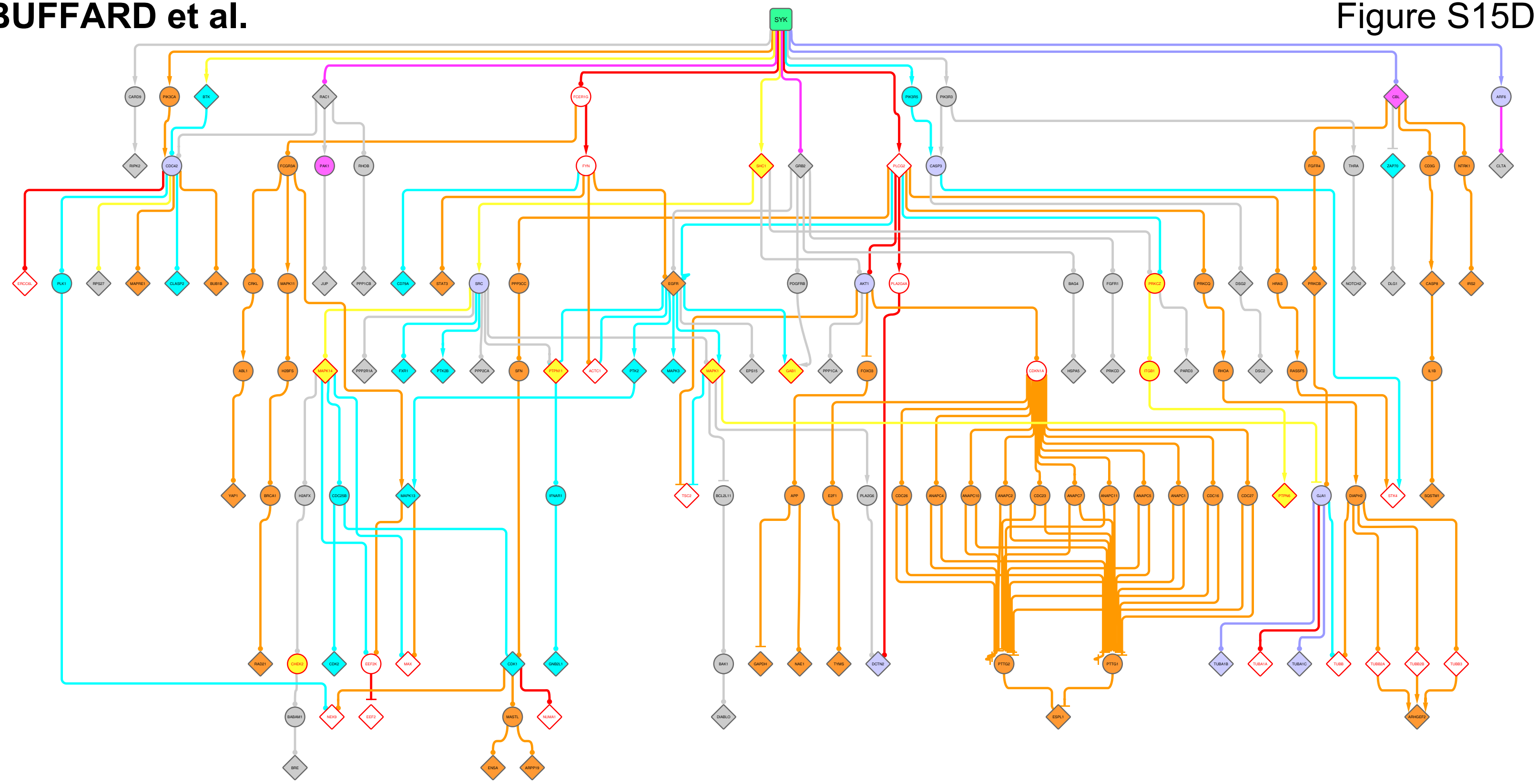

Signal propagation from Syk to its targets involved in cell growth and death. (hierarchical layout)

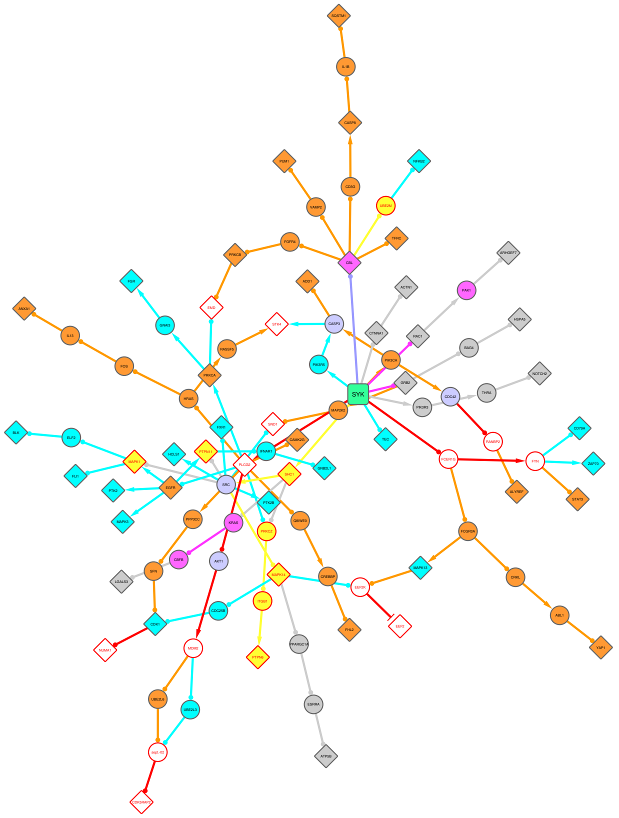

Signal propagation from Syk to its targets involved in differentiation.
(prefused force directed layout)

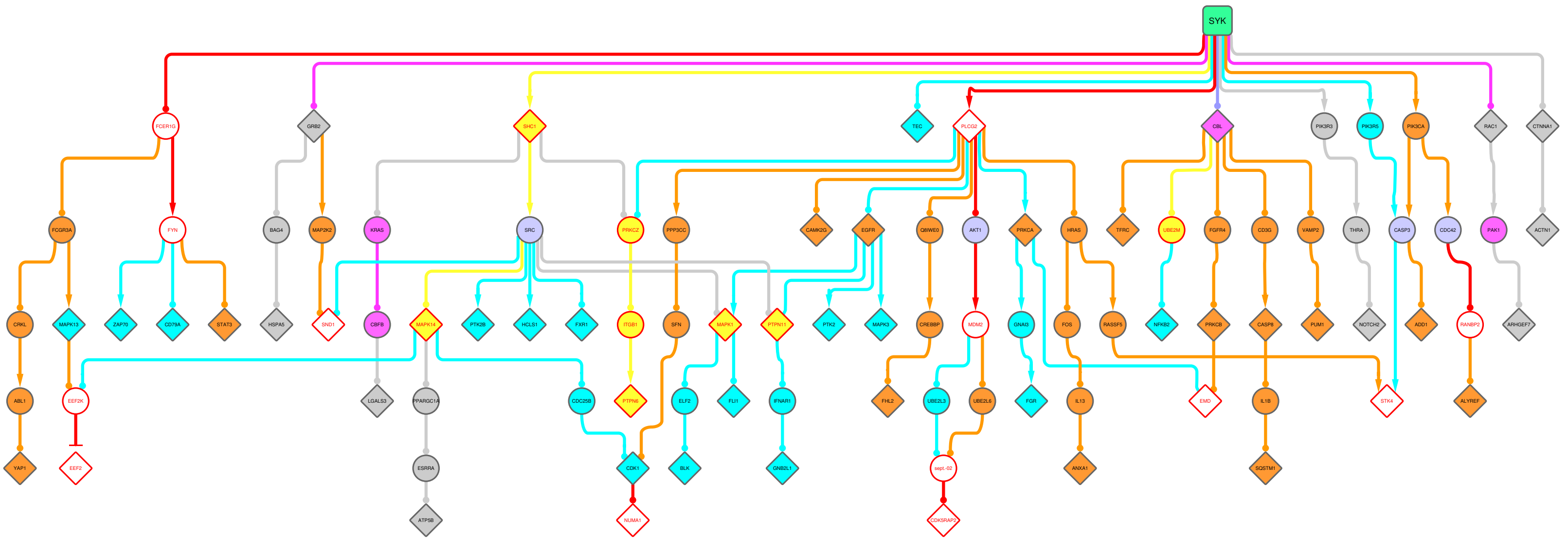

Signal propagation from Syk to its targets involved in differentiation. (hierarchical layout)

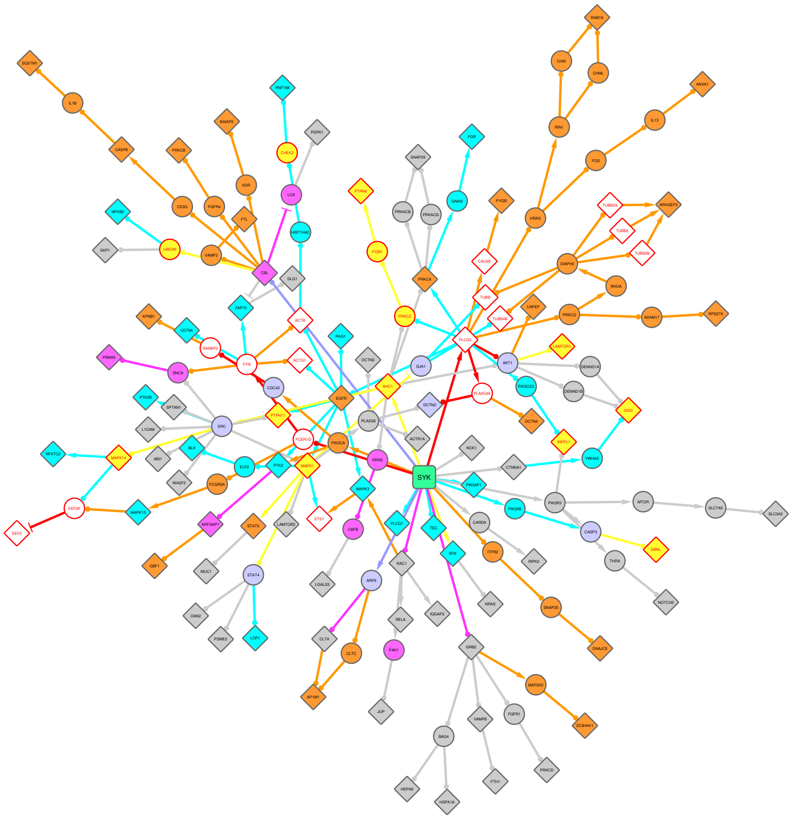

Signal propagation from Syk to its targets involved in inflammation.
(prefused force directed layout)

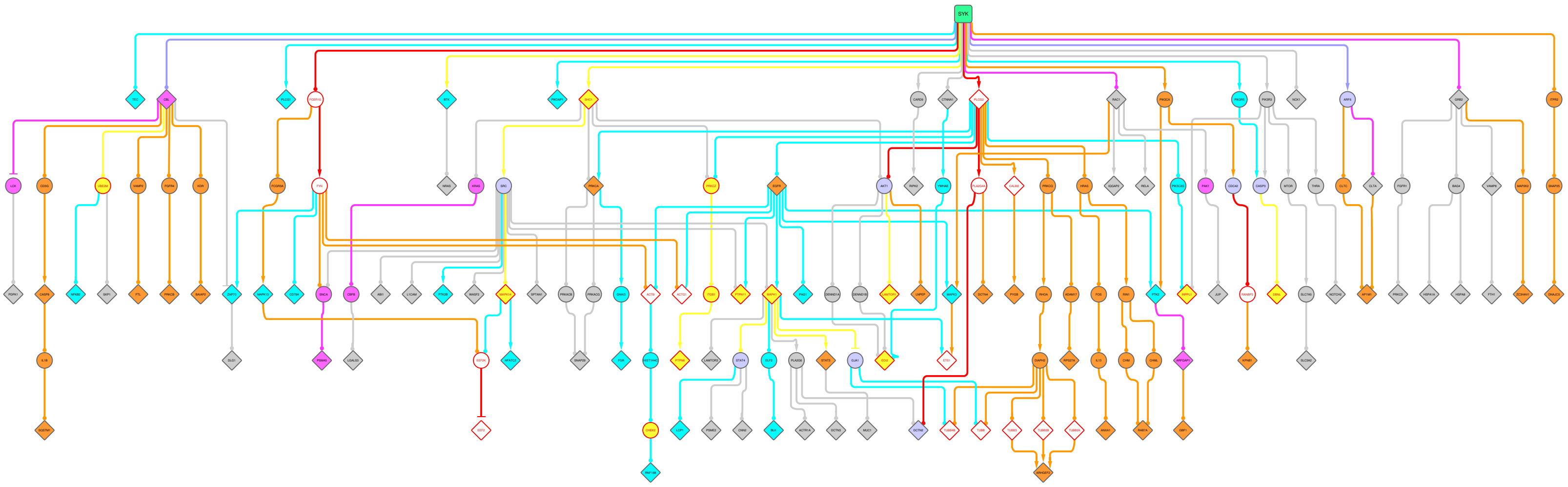

Signal propagation from Syk to its targets involved in inflammation.
(hierarchical layout)

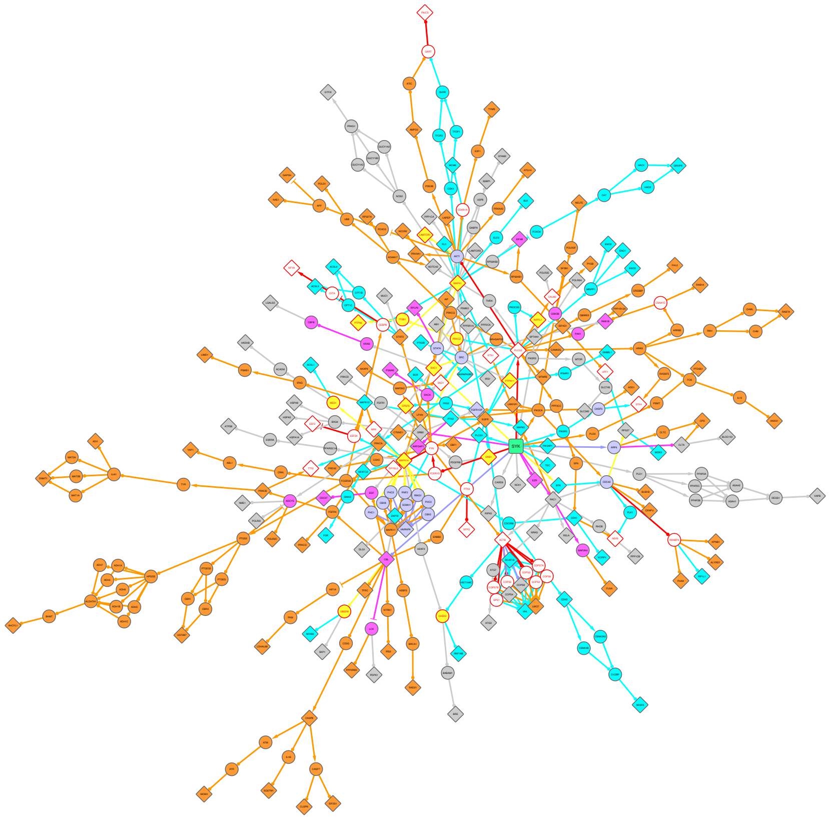

Signal propagation from Syk to its targets involved in transport and metabolism.
(prefused force directed layout)

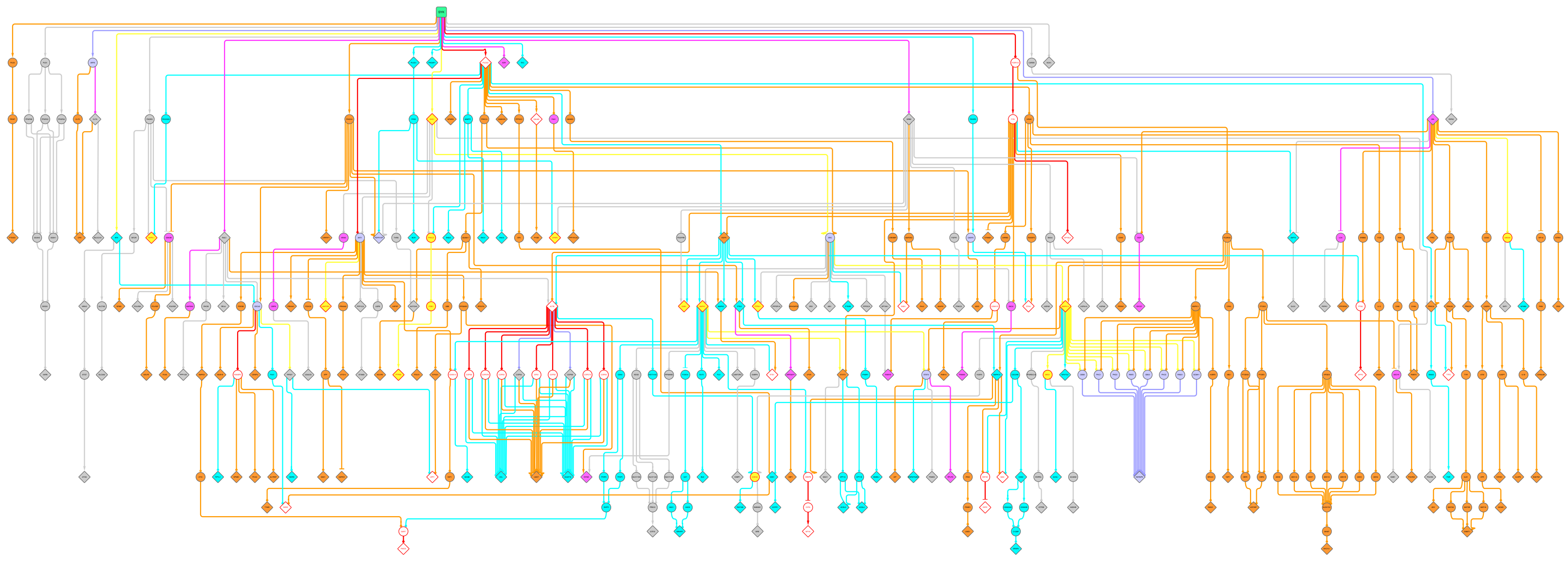

Signal propagation from Syk to its targets involved in transport and metabolism. (hierarchical layout)
